# Effect of Non- and Low-Caloric Sweeteners on Substrate Oxidation, Energy Expenditure, and Catecholamines in Humans—A Systematic Review

**DOI:** 10.3390/nu15122711

**Published:** 2023-06-11

**Authors:** Sabina S. H. Andersen, Ruixin Zhu, Louise Kjølbæk, Anne Raben

**Affiliations:** 1 Department of Nutrition, Exercise and Sports, Faculty of Science, University of Copenhagen, 1958 Frederiksberg C, Denmark; 2 Clinical Research, Copenhagen University Hospital—Steno Diabetes Center Copenhagen, 2730 Herlev, Denmark

**Keywords:** non-caloric sweeteners, low-caloric sweeteners, fat oxidation, carbohydrate oxidation, energy expenditure, catecholamines, energy homeostasis, ventilated hood, respiration chamber

## Abstract

The use of non- and low-caloric sweetener(s) (NCS and LCS) as a means to prevent overweight and obesity is highly debated, as both NCS and LCS have been proposed to have a negative impact on energy homeostasis. This systematic review aimed to assess the impact of NCS and LCS on fasting and postprandial substrate oxidation, energy expenditure, and catecholamines, compared to caloric sweeteners or water, across different doses and types of NCS and LCS, acutely and in the longer-term. A total of 20 studies were eligible: 16 studies for substrate oxidation and energy expenditure and four studies for catecholamines. Most studies compared the acute effects of NCS or LCS with caloric sweeteners under non-isoenergetic conditions. These studies generally found higher fat oxidation and lower carbohydrate oxidation with NCS or LCS than with caloric sweeteners. Findings for energy expenditure were inconsistent. With the limited number of studies, no convincing pattern for the remaining outcomes and comparisons could be seen. In conclusion, drinks or meals with NCS or LCS resulted in higher fat and lower carbohydrate oxidation compared to caloric sweeteners. No other conclusions could be drawn due to insufficient or inconsistent results. Further studies in this research field are warranted.

## 1. Introduction and Background

From 1975 to 2016, the prevalence of obesity almost tripled [1]. Obesity increases the risk of non-communicable diseases, such as type 2 diabetes (T2D) and cardiovascular diseases (CVD), which is of major public concern [1]. One strategy promoted to tackle the obesity epidemic is to reduce the consumption of foods and drinks with added sugar, as these often have low nutritional value and mainly provide energy [2]. Reducing the consumption of sugary products has led to an increase in the consumption of foods and drinks with non-caloric sweetener(s) (NCS) and low-caloric sweetener(s) (LCS), allowing a variety of products to retain their palatability without the associated energy and glycemic impact [3,4,5,6,7,8].

Non-caloric sweeteners are food additives with high-intensity sweetening properties containing no or negligible amount of energy [5,7]. Common NCS are aspartame, sucralose, saccharin, acesulfame potassium, and steviol glycosides [5,7]. Non-caloric sweeteners are used as substitutes for added sugar in both soft drinks and solid foods, such as desserts [5,9]. When NCS are added to solid foods, this is often in combination with LCS [4]. Low-caloric sweeteners are food additives that provide sweetness combined with a reduced energy content (on average 7.7 kJ/g) compared to regular caloric sweeteners, such as sucrose, glucose, and fructose (16.8 kJ/g) [4,5,10]. Common LCS are isomalt, lactitol, maltitol, xylitol, sorbitol, and erythritol—all of which are sugar alcohols [4,10]. These compounds are mainly used as substitutes for added sugar in solid foods, such as desserts, candy, etc., as they have a bulk sweetener capacity [4,10]. Diverse regulatory agencies consider both NCS and LCS to be safe for human consumption [5,7,10,11]. However, these agencies focus mainly on toxicity and carcinogenicity [7,12,13], and whether NCS and LCS are means of preventing obesity is still highly debated.

Many observational and animal studies find an increased risk of obesity, CVD, and T2D in relation to the intake of NCS [14,15,16,17,18], whilst RCTs largely do not support these findings or find the opposite [14,17,18]. This could be an indication that observational studies may be influenced by confounding factors and reverse causality—both known methodological issues for observational studies [19]. Furthermore, transferring results from animal studies to humans should be done with caution due to factors, such as physiological and/or large dose differences between animal and human studies. It is important to consider these methodological issues when evaluating the strength of evidence within this or any other area of research. That the strength of evidence within this research area is not adequately discussed was confirmed in the citation network analysis performed by Normand et al. [20]. They found that reviews concluding a beneficial relationship between NCS and body weight cited mainly RCTs, whereas reviews concluding an adverse relationship cited mainly observational studies [20], thus identifying an important risk of citation bias. This issue is clearly highlighted in the newly published recommendation by the World Health Organization (WHO) [21]. Here, the WHO advises against the use of NCS for weight management or reducing the risk of non-communicable diseases [21]. This recommendation draws upon a systematic review from 2022, which included both RCTs and observational studies—the RCTs showing decreasing effects, on e.g., BMI, body weight, and neutral effects, on e.g., markers for T2D, while the observational studies showed an increase in those outcomes [14]. In the recommendation, the RCTs and observational studies seem to have been weighted equally. In contrast to the recommendation by WHO, a 2020 expert panel’s consensus statements concluded that, e.g., NCS promote weight loss, do not affect T2D, and should be considered as a strategy to reduce sugar intake [11]. In the expert panel’s consensus statements, the results of RCTs were weighted higher than the results of observational studies [11]. Due to the risk of confounding and reverse causality within the observational studies, the recommendation by WHO is conditional; however, the above shows the discrepancy when discussing the current evidence within this research area.

Low-caloric sweeteners do not seem to give rise to the same concerns as NCS, nor do they seem to be included in most observational studies. Reasons for the latter could be that (1) LCS are natural compounds found in many fruits and vegetables and (2) LCS are mainly used in solid foods. Both factors make it difficult to isolate a potential positive or negative effect of LCS per se. However, it is well known that excess intake of LCS can create gastrointestinal problems, such as bloating and diarrhea [4]. In addition to this, it has also been proposed that LCS can potentially increase body weight through some of the same mechanisms as NCS [22].

The mechanisms by which it has been proposed that NCS and/or LCS could potentially increase body weight are that they might promote impaired sensory and endocrine signaling by stimulating sweet taste receptors without providing the carbohydrate load normally associated with such stimulation and/or by disrupting the gut microbiome [9,12,22,23,24,25,26,27]. Factors that could indicate impaired sensory and endocrine signaling, such as impaired appetite and glucose regulation, have been examined in previous reviews [4,8,28,29,30,31]. Overall, no negative effects of NCS and/or LCS were observed. The role of NCS and LCS on the other side of energy balance, i.e., energy expenditure and substrate oxidation, has not yet been investigated in detail. The aim of this systematic review was to shed further light on this by assessing the effect of NCS and LCS, across different doses and types, on fasting and postprandial fat oxidation, carbohydrate oxidation, protein oxidation, energy expenditure, and catecholamines (the regulatory hormones) compared to the effects of caloric sweeteners or water.

We aimed to answer the following research questions: (1) What are the effects of consuming NCS or LCS compared to caloric sweeteners or water on acute and longer-term fat oxidation? (2) What are the acute and longer-term effects of consuming NCS or LCS compared to caloric sweeteners or water on carbohydrate oxidation, energy expenditure, and catecholamine concentration? In addition, we report adverse events when available.

## 2. Materials and Methods

### 2.1. Protocol

Based on the guidelines from the Cochrane Handbook for systematic reviews [32] and the Preferred Reporting Items for Systematic Reviews and Meta-Analysis for Protocols (PRISMA-P) [33], a protocol was registered and published at PROSPERO on 4 November 2021 and 5 December 2021, respectively. A protocol amendment, which included a link to the full search strategy and some specified and additional details, was finalized on 22 February 2022 and published by PROSPERO on 22 September 2022 (CRD42021289241). The following changes from the registered protocol occurred during the conduction of the systematic review: Originally, energy intake was included as a secondary outcome. However, as this outcome has been thoroughly evaluated in previous systematic reviews [30,31], it was decided to exclude this outcome before the data extraction process. Accordingly, the searches were re-run without search terms for energy intake. Furthermore, some extra information from the included studies was extracted, e.g., aim, statistical analysis, and time-meal interactions.

### 2.2. Outcomes

The outcomes of interest were as follows. Primary outcome—fasting and postprandial fat oxidation. Secondary outcomes—fasting and postprandial carbohydrate and protein oxidation, energy expenditure, concentration of catecholamines, and adverse events if reported in the included studies.

### 2.3. Search Strategy

The formal search for potentially eligible studies was performed by the authors S.S.H.A and R.Z on 7 December 2021. The following electronic databases were used to identify eligible studies: Web of Science, PubMed, EMBASE (via Ovid), and Cochrane Register of Controlled Trials (CENTRAL) on the Cochrane library, which includes a search on ClinicalTrials.gov. Only human studies and articles in English were included. Filters for human studies were not available on Web of Science and CENTRAL. There were no limitations with regard to publication period or location. Furthermore, the reference lists of all included studies were screened, and scientists within this field were consulted in order to identify other potentially eligible studies. See Supplementary Tables S1 and S2 for more specific details about the search strategy. Searches were re-run on 12 December 2022 to identify any newly published eligible studies.

### 2.4. Studies Included

Studies eligible for inclusion had to fulfill the following criteria: Human trials investigating acute (≤1 day) and/or longer-term (>1 day) responses to NCS and/or LCS. The studies had to include a comparator/control group, e.g., meals, drinks, or diets without NCS or LCS but with, for example, sucrose or water as a comparator. Studies comparing different doses or types of NCS and/or LCS were also included. Postprandial values, and for the longer-term studies, also fasting values at the end of the intervention, were included as available. The NCS and/or LCS had to be orally ingested, e.g., added to meals or drinks or in capsules. Studies where NCS and/or LCS were used as the control condition were included if a comparison between any of the interventions could be made in relation to the outcomes of interest. Studies were included regardless of sex and age. Criteria for body mass index (BMI) status were: Children and/or adolescents = BMI-for-age z-score > −2 standard deviations (SD). Adults = BMI ≥ 18.5 kg/m^2^. No upper limit for BMI-for-age z-score/BMI was defined.

Specific exclusion criteria were as follows: Studies including only within-group measures were excluded due to the disadvantages of this type of analysis [34]. Studies including participants with cancer (currently active diagnoses) were excluded.

### 2.5. Data Extraction

All studies found via the search strategy were uploaded to the review software Covidence [35], duplicates were removed, and S.S.H.A and R.Z independently screened titles and abstracts, read all potentially relevant articles, selected the articles to be included, extracted the relevant data, and conducted a risk of bias assessment of the included studies. In case of any doubts or disagreements, A.R and L.K were consulted. The reference lists of all included studies were screened by S.S.H.A for other relevant studies.

The following was extracted from each study: (1) General information—title, authors, year of publication, and aim. (2) Characteristics of the study—design and duration. (3) Intervention—NCS and/or LCS used, comparator, composition of meals, drinks, and/or diets. (4) Characteristics of the participants—number of participants, distribution of sex, age, BMI, health status (e.g., diabetes and CVD). (5) Outcomes—means ± SD (if possible) for fasting and postprandial fat, carbohydrate, and protein oxidation, energy expenditure, catecholamines, and adverse events. The corresponding *p*-values for the difference in means between the compared groups were also extracted, and these were used as the effect of measures. A difference between means was defined as a *p*-value < 0.05. Intention-to-treat (ITT) data were extracted, if possible. (6) Statistical analyses—type of analyses and included covariates.

It became clear that some studies did not report mean ± SD for given outcomes but instead provided time-meal interactions—making it impossible to fulfill point 5. Therefore, it was decided to extract time-meal interactions for all included studies when provided. When extracting results for mean postprandial fat and carbohydrate oxidation and energy expenditure, it was preferred to extract the results as either area under the curve (AUC), incremental area under/over the curve (iAUC/iAOC), or diet-induced thermogenesis (DIT) (corresponding to iAUC for energy expenditure). However, this was not possible for all studies. In three studies, the mean postprandial measurements were provided as kJ/min or g/min for either fat oxidation or carbohydrate oxidation [36,37,38]. Furthermore, one of these studies did not provide a relevant measurement for fat oxidation, and the respiratory quotient (RQ) was therefore extracted as a measure of fat oxidation [36]. Similarly, two studies [39,40] did not provide results for fat and carbohydrate oxidation separately but solely by RQ, which was extracted as a joint measure. The area under the curve provides information about total substrate oxidation and energy expenditure. Incremental area under the curve and DIT provide information about how much carbohydrate oxidation and energy expenditure are increased compared to fasting. Incremental area over the curve provides information about how much fat oxidation is inhibited compared to fasting, and RQ provides information about which of the two substrates is predominantly being oxidized.

### 2.6. Risk of Bias

The Cochrane Collaboration tool for assessing the risk of bias in randomized and non-randomized studies was used [41,42,43,44]. Six domains were included for RCTs: (1) Bias arising from the randomization process. (2) Bias arising from period and carryover effects (only relevant for crossover studies). (3) Bias due to deviations from the intended interventions. (4) Bias due to missing outcome data. (5) Bias in measurement of the outcome. (6) Bias in the selection of the reported results. Seven domains were included for non-RCTs: (1) Bias due to confounding. (2) Bias in the selection of participants into the study. (3) Bias in the classification of interventions. (4) Bias due to deviations from intended interventions. (5) Bias due to missing data. (6) Bias in measurement of the outcome. (7) Bias in the selection of the reported results. As the assessment was done at both outcome and study levels, one assessment per outcome for a given study was made. However, as all outcomes from a given study received the same final evaluation, a joint evaluation was reported.

### 2.7. Data Analysis

As defined in the protocol, a minimum of five comparable studies were required for a meta-analysis to be carried out. This number was chosen to ensure both power and precision. The following parameters were considered when assessing homogeneity between studies: (1) Was the study an acute study or a longer-term study? (2) How were the results provided, e.g., as AUC, iAUC, iAOC, DIT, time-meal interaction, and was an actual number or only a *p*-value reported as a result? (3) What type of sweetener (NCS or LCS) was used, how was it served, and what was it compared to? Five comparable studies were not found for any of the outcomes, and therefore evidence tables of key study characteristics were synthesized, accompanied by a narrative synthesis, following the guidelines outlined in “Synthesis without meta-analysis in systematic reviews: reporting guidelines” [45].

An evaluation of the quality of evidence for all outcomes was performed using the system Grading of Recommendations Assessment, Development, and Evaluation (GRADE) [46,47]. The evaluation includes four levels of quality: (1) High quality—further research is very unlikely to change our confidence in the estimate of effect. (2) Moderate quality—further research is likely to have an important impact on our confidence in the estimate of effect and may change the estimate. (3) Low quality—further research is very likely to have an important impact on our confidence in the estimate of effect and is likely to change the estimate. (4) Very low quality—any estimate of effect is very uncertain. When defining the quality of evidence for a given outcome, the quality of the studies investigating this outcome was evaluated based on the following five domains: (1) Study limitations. (2) Inconsistency of results. (3) Indirectness of evidence. (4) Imprecision. (5) Reporting bias. Evidence from RCTs always begins as high-quality evidence but can decrease in the level of quality in relation to these five domains, and vice versa for non-RCTs.

## 3. Results

### 3.1. Search Results

**Figure 1:**
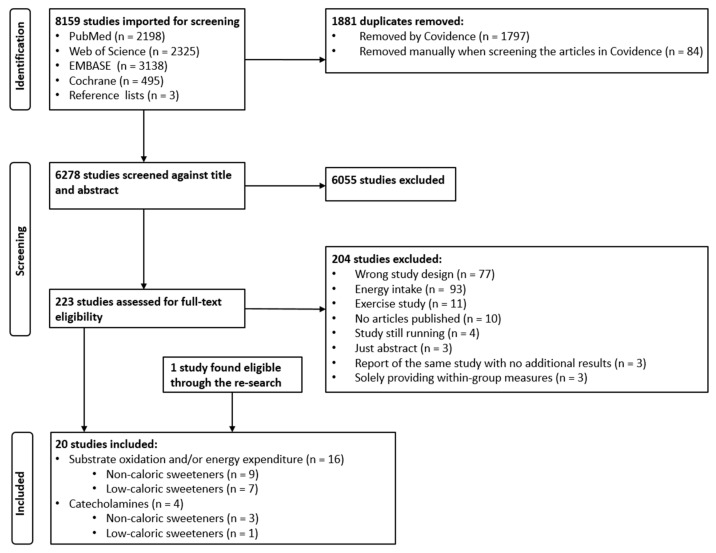
PRISMA flow chart showing the selection process of the studies.

The formal search performed on 7 December 2021 resulted in a total of 8156 studies, and three more studies were found via reference lists. After the removal of duplicates, a total of 6278 studies were screened via title and abstract. Of these, 223 studies were deemed eligible for full-text assessment. The rest were excluded due to being reviews, animal or observational studies, or having no relevance to our outcomes/topic, etc., despite relevant search words in the title or abstract. After the full-text assessment, 22 studies were included. Three of these studies [48,49,50] were then excluded because they only provided within-group measures. One new eligible study was found when the search was re-run on 12 December 2022. Thus, a total of 20 studies were included. Full-text screening found 11 studies focusing on exercise performance (e.g., measured through substrate oxidation and energy expenditure) after ingestion of NCS (control group) or some type of carbohydrate. These studies were excluded, as the exercise component would mask the effect of NCS per se. See Figure 1 for more details.

### 3.2. Study Characteristics—All Studies

Overviews of the study characteristics and results are presented in Table 1, Table 2 and Table 3. A more comprehensive presentation of the studies can be found in Supplementary Tables S3–S5. Of the 20 included studies [36,37,38,39,40,51,52,53,54,55,56,57,58,59,60,61,62,63,64,65], 18 were RCTs, whereof 17 had a crossover design [36,37,38,39,40,51,53,55,56,58,59,60,61,62,63,64,65] and one a parallel design [52]. The last two studies were non-RCTs with a crossover design [54,57].

Eighteen studies included adult participants only [36,37,38,39,40,51,52,53,54,55,56,58,59,61,62,63,64,65], one study included children only [60], and one study included both children and adults [57], but here only the results for the children were of relevance. Thirteen studies included both sexes [36,40,51,52,53,54,57,58,59,60,61,62,63], six studies included only males [37,38,55,56,64,65] and one study did not provide any information about sex [53]. Ten studies included participants with normal weight [38,40,51,53,55,56,59,61,63,64], five studies included participants with both normal and overweight [36,37,58,62,65], one study included participants with overweight only [52], and four studies [39,54,57,60] did not include information about weight classification. No studies included participants with diabetes or CVD (Table 1, Table 2 and Table 3, Supplementary Tables S3–S5).

A total of 12 studies included NCS [36,37,51,52,57,58,60,61,62,63,64,65], five studies with sucralose [36,37,51,61,62], four studies with aspartame [58,60,64,65], one study with aspartame and d-allulose [63], one study with aspartame and acesulfame potassium [57], and one study with a mix of NCS (54% aspartame, 23% cyclamate, 22% acesulfame potassium k, and 1% saccharin) [52]. Five studies compared a NCS to a caloric sweetener: sucrose, glucose, and/or fructose [36,37,52,57,61]. Two studies included NCS mixed with maltodextrin and compared this to sucrose, maltodextrin, or to the NCS alone [62,64]. Three studies included a non-caloric substance—water or microcrystalline cellulose—as the only comparator or in addition to a caloric sweetener condition [58,60,65]. One study was a dose-response study [51] and one study compared two different types of NCS [63]. Four studies tested solely non-isoenergetic conditions [36,52,57,61], six studies solely isoenergetic conditions [37,51,58,60,63,64], and two studies both a non-isoenergetic and an isoenergetic condition [62,65]. Only one study provided a fasting measure of relevance—basal metabolic rate (BMR) after 10 weeks of regular exposure to a mix of NCS [52] (Table 1 and Table 3, Supplementary Table S3).

A total of eight studies included LCS [38,39,40,53,54,55,56,59], presenting a range of LCS, maltitol [53], mannitol and xylose [59], xylitol [54], lactitol [39], xylitol and lactitol [38], isomalt [55], d-tagatose [40], high-polymer maltitol syrup (50% maltitol and 50% polymer), and the polymer fraction alone [56]. Seven studies compared the LCS to a caloric sweetener; sucrose, glucose, or dextrose [38,39,40,53,54,55,56], and one study compared the LCS to water [59]. All these studies were non-isoenergetic. Only postprandial results were provided for both acute and longer-term studies (Table 2 and Table 3, Supplementary Table S4).

The NCS and LCS were either served in a drink, capsules, or some type of solid food. When in a drink, this was served either alone or in combination with a standardized meal/meals. In non-isoenergetic studies, the energy difference between interventions was caused by the extra carbohydrate load of the caloric sweetener compared to the NCS and LCS. In isoenergetic studies, the NCS was either compared to a non-caloric substance or the carbohydrate load was matched across interventions with e.g. maltodextrin (Table 1, Table 2 and Table 3).

### 3.3. Fat Oxidation

A total of 12 studies provided results for our primary outcome fat oxidation—seven studies for NCS [36,37,52,61,62,63,65] (Table 1, Supplementary Table S3), and five studies for LCS [38,39,40,53,55] (Table 2, Supplementary Table S4).

#### 3.3.1. Non-Caloric Sweeteners

Five studies found a higher fat oxidation after NCS compared to sucrose, glucose, fructose, and/or glucose/fructose under non-isoenergetic conditions [36,52,61,62,65], whereas one study found no difference in fat oxidation after jelly with sucralose compared to jelly with sucralose and maltodextrin [62]. Under isoenergetic conditions, one study observed no difference in fat oxidation [37] while one study found a higher fat oxidation after jelly with sucralose and maltodextrin compared to jelly with sucrose [62]. Another study [65] found no difference in total fat oxidation between aspartame and water. However, fat oxidation was lower 10 min after aspartame compared to water [65]. In the study comparing two different NCS, a higher fat oxidation was seen after d-allulose compared to aspartame [63] (Table 1).

#### 3.3.2. Low-Caloric Sweeteners

Three studies found a higher fat oxidation after either xylitol, isomalt, or lactitol compared to glucose or sucrose [38,39,55]. In contrast, two studies found no difference in fat oxidation after lactitol or d-tagatose compared to glucose or sucrose [38,40]. Lastly, one study found that fat oxidation was higher after maltitol in the first part of the postprandial phase compared to sucrose [53] and the opposite was seen at the end of the postprandial phase (Table 2).

### 3.4. Carbohydrate and Protein Oxidation, Energy Expenditure, Catecholamines and Adverse Events

A total of 20 studies provided results for the secondary outcomes, 12 studies for NCS [36,37,51,52,57,58,60,61,62,63,64,65] (Table 1 and Table 3; Supplementary Tables S3 and S5) and eight studies for LCS [38,39,40,53,54,55,56,59] (Table 2 and Table 3; Supplementary Tables S4 and S5). Not all studies provided results for all secondary outcomes. Due to the reciprocal relationship between fat and carbohydrate oxidation [66], a majority of the results for carbohydrate oxidation match the results, though in the opposite direction, for fat oxidation. Therefore, only results not following this pattern are described in the following.

#### 3.4.1. Non-Caloric Sweeteners

Carbohydrate oxidation: A total of seven studies provided results for carbohydrate oxidation [36,37,52,61,62,63,65], and five of these displayed the above mentioned reciprocal pattern between fat and carbohydrate oxidation. One non-isoenergetic study found a lower carbohydrate oxidation after jelly with sucralose compared to jelly with sucralose and maltodextrin [62], and one isoenergetic study found a higher carbohydrate oxidation after sucralose compared to sucrose [37] (Table 1).

Protein oxidation: Only two studies provided results for protein oxidation—both studies were non-isoenergetic [52,61]. One study found a higher protein oxidation after sucralose compared to sucrose [61], and one study found no difference in protein oxidation after a diet/meal with NCS compared to sucrose [52] (Supplementary Table S3).

Energy expenditure: A total of eight studies measured energy expenditure [37,51,52,61,62,63,64,65]. Under non-isoenergetic conditions, total energy expenditure (i.e., AUC) was lower after sucralose compared to sucrose in two studies [61,62], whilst DIT was significantly higher after sucralose compared to sucrose in one of these studies [61]. In contrast, no difference for total energy expenditure and DIT was found in two other studies comparing NCS to sucrose [52,65]. Lastly, no difference in BMR was seen after a 10-week intervention including a diet with NCS compared to sucrose [52]. Under isoenergetic conditions, the two studies [37,62] comparing sucralose combined with some type of carbohydrate to sucrose found no difference in total energy expenditure, whilst one of these studies found a higher DIT after sucralose compared to sucrose [37]. One study found a lower energy expenditure at specific time points after aspartame with maltodextrin compared to sucrose [64], while no difference was found when comparing aspartame with maltodextrin to maltodextrin alone. This study found no difference in DIT when comparing aspartame with maltodextrin to sucrose, or to maltodextrin [64]. Aspartame resulted in a higher total energy expenditure compared to water in one study [65], and one study found no difference between d-allulose and aspartame [63]. In the study testing different doses of NCS, DIT was found to be lower after the lowest concentration compared to the highest concentration of sucralose [51] (Table 1).

Catecholamines: A total of three studies provided results for catecholamines [57,58,60]. All studies measured epinephrine, two studies measured norepinephrine [58,60], and one study measured dopamine [60]. The study comparing aspartame and acesulfame potassium to sucrose in non-isoenergetic conditions found that epinephrine concentration was lower after aspartame and acesulfame potassium at specific time points [57]. The two studies comparing aspartame to microcrystalline cellulose in isoenergetic conditions found no differences in epinephrine, norepinephrine, or dopamine concentrations [58,60] (Table 3).

Adverse events: No adverse events were found for NCS in the three studies that declared this information [36,58,60] (Supplementary Tables S3 and S5).

#### 3.4.2. Low-Caloric Sweeteners

Carbohydrate oxidation: A total of six studies provided results for carbohydrate oxidation [38,39,40,53,54,55]—i.e., one more study than for fat oxidation. Five of these studies showed results fitting the reciprocal pattern between fat and carbohydrate oxidation [38,39,40,53,55], whilst one showed a result deviating from the pattern [38]. One of the studies also provided results for iAOC in addition to a time-meal interaction [53]. One study found no difference in carbohydrate oxidation when comparing xylitol to glucose [38], one study found a lower carbohydrate oxidation when comparing maltitol to sucrose [53], and one study found a 23% lower carbohydrate oxidation after xylitol compared to glucose [54], but a significance level was not stated (Table 2).

Protein oxidation Only one study provided results for protein oxidation [40]. No difference in protein oxidation was found after d-tagatose compared to sucrose (Supplementary Table S4).

Energy expenditure: A total of three studies provided results for energy expenditure [40,54,56]. One study found a 46% lower total energy expenditure after xylitol compared to sucrose (significance level not stated) [54], one study found a higher total energy expenditure after high-polymer maltitol syrup and the polymer fraction alone compared to dextrose [56], whereas one study found no difference in total energy expenditure after d-tagatose compared to sucrose [40] (Table 2).

Catecholamines: Only one study provided results for catecholamines [59], and here no differences in epinephrine concentrations were seen after xylose and mannitol compared to water (Table 3).

Adverse events: Mild gastrointestinal symptoms, e.g., excessive gas emission and frequent stool, were noted for LCS in the three studies reporting information about adverse events [40,54,56] (Supplementary Table S4).

### 3.5. Risk of Bias Assessment

**Figure 2:**
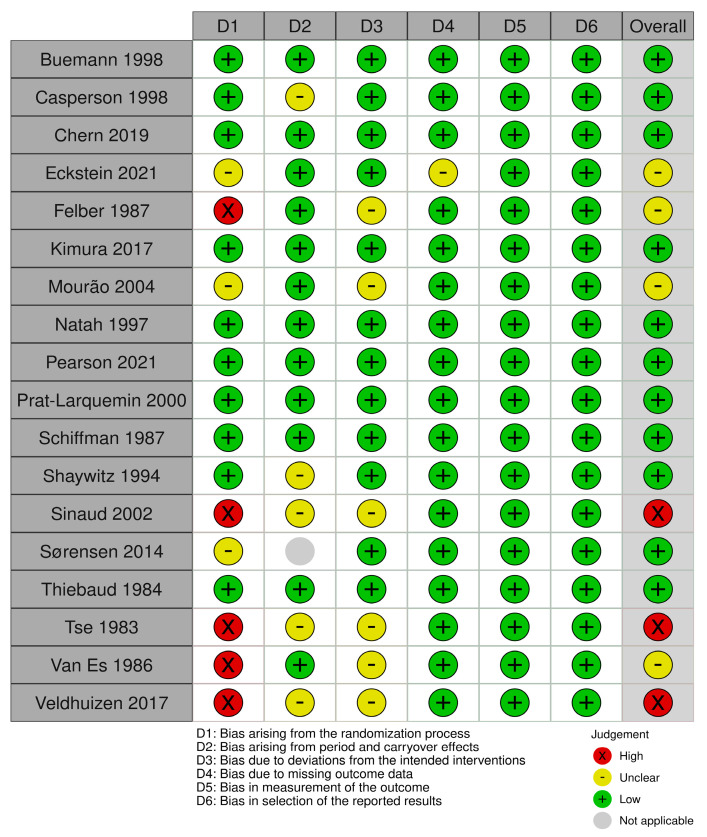
Risk of bias assessment for randomized controlled trials. The Cochrane Collaboration tool for assessment of risk of bias in randomized trials was used [36,37,38,39,40,41,42,44,51,52,53,55,56,58,59,60,61,62,63,64,65].

**Figure 3:**
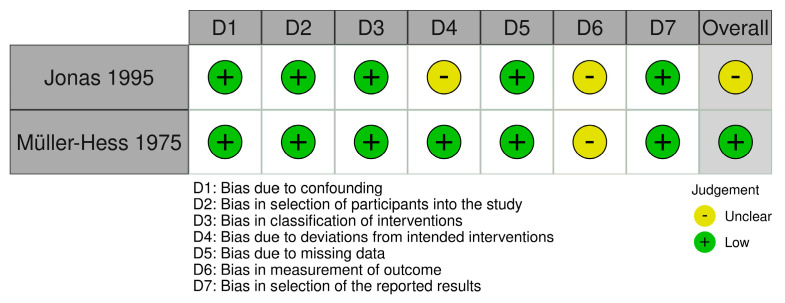
Risk of bias assessment for controlled non-randomized trials. The Cochrane Collaboration tool for assessment of risk of bias in non-randomized trials was used [43,54,57].

In total, 12 studies received a low overall risk of bias score (RoB), [38,40,52,54,55,58,60,61,62,63,64,65], five studies an unclear RoB [36,37,39,53,57] and three studies a high RoB [51,56,59]. The RCTs received a variation of low, unclear, or high scores across the domains D1–D4 (Figure 2). It was not possible to determine whether a randomization of the participants actually occurred in the five studies receiving a high score in D1, but based on the study descriptions, we assumed these studies to be RCTs [39,51,53,56,59]. Both non-RCTs received an unclear score for domain D6, which concerns, among other things, blinding [54,57] (Figure 3).

## 4. Discussion

### 4.1. Summary of Findings

Twelve studies included NCS, primarily representing aspartame and sucralose, while eight studies included LCS, encompassing a broader range of LCS, namely, maltitol, mannitol, xylose, xylitol, lactitol, isomalt, and d-tagatose. Most studies found a higher fat oxidation and lower carbohydrate oxidation after intake of NCS or LCS compared to caloric sweeteners under non-isoenergetic conditions. Results for total energy expenditure and DIT were inconsistent, showing both lower, higher, or no differences after NCS or LCS compared to caloric sweeteners under non-isoenergetic conditions. With the limited number of studies, no convincing pattern for the remaining outcomes and comparisons could be seen. Low-caloric sweeteners were only compared to water for the outcome catecholamines, and no studies provided results comparing LCS to caloric sweeteners under an isoenergetic condition. Primarily, acute studies were represented among all outcomes. No adverse events were found for NCS, but mild gastrointestinal symptoms, such as excessive gas and frequent stool, were reported for LCS in the limited number of studies reporting adverse events.

### 4.2. Fat and Carbohydrate Oxidation

Due to the reciprocal relationship between fat and carbohydrate oxidation [66], these outcomes are discussed together. The carbohydrate content of the meal is recognized as one of the main determinants influencing fat and carbohydrate oxidation during the postprandial phase [66,67,68,69]. However, fuel utilization is influenced by various other factors, including but not limited to age, training status, exercise, the preceding meal, and body composition [68,69,70,71]. In many of the included studies, such factors are adequately controlled for by using randomized controlled crossover designs and standardizing, e.g., exercise and food intake in the day leading up to the test days. This approach ensures that the impact of these factors is minimized on the outcomes of interest. Thus, the higher fat and lower carbohydrate oxidation observed in most studies comparing NCS and LCS to caloric sweeteners under non-isoenergetic conditions are most likely caused by the higher carbohydrate content in the drinks/meals containing the caloric sweeteners. The higher carbohydrate content will cause increased insulin concentrations and hence inhibit fat oxidation and stimulate carbohydrate oxidation compared to NCS and LCS [66,67,68]. This assumption is confirmed by the study comparing NCS to water under isoenergetic conditions, as no difference in effect on fat and carbohydrate oxidation was seen [65]. The lack of effect of NCS and LCS on fat and carbohydrate oxidation is supported by a newly published systematic review showing no effect of NCS on, e.g., insulin [29] and the overall low insulinemic properties of LCS [8]. Only a few results contradicted this interpretation, and here methodological issues may have influenced the results.

Looking at studies including NCS, Chern et al. [62] found a higher fat oxidation and lower carbohydrate oxidation after jelly with sucralose and maltodextrin compared to jelly with sucrose (isoenergetic condition). One could ascribe this to the fact that sweet taste receptors were stimulated by two different types of sweeteners: sucralose and sucrose. However, another explanation could be the differences between the metabolism of maltodextrin and sucrose combined with the short duration of the postprandial measurements (1.5 h). Maltodextrin and sucrose are both thought to give a rapid glycemic response [72]. This makes maltodextrin (a non-sweet carbohydrate) an ideal choice when testing whether NCS combined with a simple carbohydrate affects fat and carbohydrate oxidation differently than sucrose. However, sucrose will, in contrast to maltodextrin, stimulate sweet taste receptors throughout the gastrointestinal track, beginning in the oral cavity [73]. A stimulation of the sweet taste receptors raises, among other things, concentrations of glucagon-like peptide-1 (GLP-1), and glucose-dependent insulinotropic peptide (GIP), which in turn upregulate the sodium glucose transporter (SGLT1) and glucose transporter 2 (GLUT2) [73]. This will stimulate the uptake of glucose [73]. Maltodextrin cannot stimulate the sweet taste receptors before it has been broken down into glucose. The reduced stimulation of the sweet taste receptors by maltodextrin compared to sucrose in the gastrointestinal track might explain the differences between the two jellies, since sucrose would presumably give a more rapid uptake of glucose early in the postprandial phase. Support for this interpretation of the results is found in the study by Prat-larquemin et al. [64]. Here, no difference in DIT was found between any of the interventions (isoenergetic condition). However, both the cheese with aspartame and maltodextrin and the cheese with maltodextrin alone gave a lower postprandial energy expenditure from 0.5–1 and 1.5–2 h, and lower peak values, compared to cheese with sucrose. Furthermore, no difference was found between the cheese with aspartame and maltodextrin and the cheese with maltodextrin alone. This indicates that the differences at these time points are due to differences in the metabolism of maltodextrin and sucrose. The above highlights that if maltodextrin is used to standardize the carbohydrate content across interventions, a control-group should be included, where maltodextrin is served without the NCS. The higher carbohydrate oxidation after the meal with sucralose compared to the meal with sucrose in the isoenergetic study by Mourão et al. [37] might also be explained by challenges with standardization across interventions. In this study, the intervention meals are described as being standardized for macronutrients and energy, but the meal with sucralose had a much larger volume, a more complex macronutrient composition and the protein content seemed larger—all of which could explain the results. Another methodological issue is the relatively small sample size (*n* = 11) seen in the study by Chern et al. [62].

In studies investigating LCS, a small sample size and short duration of measuring fat and carbohydrate oxidation might also be part of the explanation for the lack of differences between interventions in the non-isoenergetic study by Natah et al. [38]. Furthermore, an important methodical issue for studies including LCS is the production of short chain fatty acids (SCFA) [74]. Short chain fatty acids are produced by the fermentation of LCS in the colon, which can cause an extra production of CO_2_ and, thus, an overestimation of carbohydrate oxidation [74]. Adjustment of the above can be made by measuring H_2_ and methane, although exact adjustment may be difficult [74]. This methodological issue could explain the lack of difference in the 24-h RQ between d-tagatose and sucrose in the study by Buemann et al. [40]. In this study, an adjustment was not made [40], whereas an adjustment was presumably made in the study by Van Es et al. [39]. In the latter, 24-h RQ was lower after lactitol compared to sucrose [39], supporting the methodological issue in relation to the study by Buemann et al. [40].

In summary, it is likely that these methodological issues could be the cause of the few contrasting results found for fat and carbohydrate oxidation.

### 4.3. Energy Expenditure

Previous studies have found that alongside protein content, the most dominate factor determining the increase in energy expenditure after a meal is the energy content of the meal [75]. Thus, the intervention meals containing most energy would presumably give the highest total energy expenditure and DIT, and there would be no difference between isoenergetic comparisons. However, this pattern was not observed in many of the studies. Again, methodological issues need to be discussed.

Looking at studies including NCS several issues seem apparent. In the isoenergetic study by Mourão et al. [37], the lack of standardization between the intervention meals is a likely explanation for the higher DIT in the sucralose group compared to the sucrose group. Another standardization issue is the timing and standardization of the meal consumed on the day prior to measuring BMR. Overfeeding has been shown to elevate BMR up to 14 h after the last meal [76], which could cause an underestimation of DIT if BMR is measured too close to the last meal. In addition to this, it has previously been shown that DIT should preferably be measured for ≥5 h and that DIT can increase with up to 7% if measured up to 8 h, an increase correlated with meal size [77]. All of the above might explain the lack of difference in DIT in the study by Sørensen et al. [52], as BMR was measured 12.5 h after an *ad libitum* meal, where the sucrose group consumed almost 1 MJ more than the NCS group, and DIT was only measured for 4 h after a meal where the sucrose group again consumed more energy than the NCS group. Other methodological issues are the small sample size (*n* = 8) in the study by Pearson et al. [65], and the high RoB assessment in the study by Veldhuizen et al. [51]—both factors create uncertainty about the otherwise novel findings of these two studies.

For LCS, the metabolism of SCFA might have affected the results of the two longer-term studies, which found no difference in energy expenditure after lactitol or d-tagatose compared to sucrose [39,40]. Short chain fatty acids have been shown to increase fat oxidation and hereby energy expenditure [78]. Thus, the fermentation of LCS in the colon generally creates difficulties when assessing the effect of LCS on substrate oxidation and energy expenditure. This issue is primarily a problem when measuring the outcomes over several hours [79,80].

In summary, these methodological issues might be part of the explanation for the contrasting results seen for energy expenditure. However, they do not explain all of these contrasts—especially for NCS. Furthermore, there was no indication that facultative thermogenesis was a factor in energy expenditure in the interventions including NCS or LCS. However, the evidence is scarce.

### 4.4. Possible Mechanism for NCS and LCS

In addition to methodological issues, the effects of NCS and LCS on substrate oxidation and energy expenditure should be considered as possible explanations for the observed deviations from the presumed primary determinants of these outcomes [66,67,75].

Sweet taste receptors are found in various locations, including the oral cavity, intestines, pancreas, brain, and adipose tissue [73]. They are thought to perform a role in glucose sensing, satiety hormone secretion, and glycemic control [73]. If NCS and LCS can stimulate sweet taste receptors beyond providing a sweet taste, it may acutely suppress fat oxidation and increase glucose oxidation and energy expenditure, mimicking the effect of a carbohydrate load. Looking at long term effects, repeated stimulation of the cephalic-phase responses (CPRs) by NCS has been suggested to impair glucose metabolism by suppressing the response to a carbohydrate load [9,24,25,26], potentially leading to inadequate inhibition of fat oxidation and insufficient increase in glucose oxidation and energy expenditure. Additionally, repeated use of NCS and/or LCS may induce chronic low-grade inflammation affecting fat disposition, insulin resistance, and energy metabolism through gut microbiota dysbiosis [22,23]—again potentially affecting substrate oxidation and energy expenditure as above. Although it is difficult to determine whether these effects could have influenced the results of the included studies, it cannot be dismissed, based on current evidence, that the deviating results, particularly in energy expenditure, may be partly explained by some of these factors. However, it is important to note that the evidence supporting these mechanisms primarily comes from animal or in vitro studies, and RCTs generally do not support these findings [9,12,22,23,24,25,26]. Lastly, it is important to note that not all of the mechanisms mentioned above may be relevant for a specific NCS or LCS, as it depends on their individual metabolism.

### 4.5. Strength of the Evidence—GRADE

The quality of evidence was low for fat and carbohydrate oxidation when comparing NCS to caloric sweeteners under non-isoenergetic conditions. The studies were all RCTs, they received a low RoB score, and the results were overall consistent across studies and type of NCS. However, it was not possible to perform a meta-analysis and estimate an effect size, so a higher quality of evidence could not be given. The quality of evidence for energy expenditure under these conditions was deemed very low. This was primarily due to the inconsistency of results between studies, lack of effect size, and limited number of studies.

The quality of evidence was also low for fat and carbohydrate oxidation when comparing LCS to caloric sweeteners under non-isoenergetic conditions. All studies except one were RCTs and the results were consistent across studies and type of LCS overall. However, as an effect size could not be estimated, the RoB score was mixed across studies, and because of the relatively small number of studies, a higher quality of evidence could not be given. The quality of evidence for energy expenditure was deemed to be very low. This was primarily due to the lack of effect size and the very limited number of studies.

The quality of the evidence was deemed very low for the rest of the study conditions and outcomes, due to the limited number of studies.

### 4.6. Limitations and Strengths

To the best of our knowledge, this systematic review is the first of its kind mapping these specific outcomes in relation to intake of NCS and LCS. Thus, a strength of this review is that it gives new insights into this research area both with regard to providing new evidence, but also by showing the pitfalls when evaluating these outcomes. Other strengths of the review are the extensive literature search, thorough systematic evaluation of each study, detailed presentation of the results, and evaluation of evidence by GRADE.

A limitation of the review is that we were not able to perform any meta-analyses because the parameters used to assess homogeneity only allowed a maximum of three studies to be included in a meta-analysis. Another limitation was the rather small sample size in many of the studies. The small sample sizes limit the precision of the estimates, increase uncertainty of the findings and make it difficult to generalize the results to a broader population. Thus, it cannot be ruled out that inconsistency between studies is simply due to lack of power. Other limitations of the included studies that could potentially have affected the results are the general issues observed in the Rob assessment. Here common problems were lack of information about the randomization process or blinding in the RCTs, or simply lack of blinding, and risk of a carryover effect in the crossover studies. Lastly, no other limits than human studies and English language were defined when running our search. This approach was intended to minimize the risk of overlooking any relevant studies. However, this approach also meant that a large number of studies had to be screened manually, which could increase the possibility of errors during the screening. To prevent errors in the screening process, the filter tools provided in Covidence were used, enabling the swift removal of numerous irrelevant studies, e.g., observational studies, reviews, animal studies, etc. [35].

## 5. Conclusions

In conclusion, drinks or meals with NCS or LCS resulted in higher fat and lower carbohydrate oxidation compared to caloric sweeteners under non-isoenergetic conditions. Results for total energy expenditure and DIT were inconsistent, showing both lower, higher, or no differences after NCS or LCS compared to caloric sweeteners under non-isoenergetic conditions. With the limited number of studies, no convincing pattern for the remaining outcomes and comparisons could be seen. The primary strength of evidence for all outcomes lies within an acute setting. More studies with focus on proper standardization are warranted for all outcomes and conditions in order to enable meta-analyses in the future.

## Figures and Tables

**Table 1:** Non-caloric sweeteners—fat oxidation, carbohydrate oxidation, and energy expenditure. Unless otherwise stated, all outcomes were measured via a ventilated hood mask, and the studies had a randomized crossover design.

First Author, Year	Population Characteristics	Intervention and Control	Results		
			FAT	CHO	EE, DIT and BMR
**Acute studies ≤ 1 day**					
Casperson, 2017 [61]	n: 27 (f = 14, m = 13) f: BMI: 22.2 ± 2 kg/m^2^ Age: 24 ± 7 y m: BMI: 24.2 ± 2 kg/m^2^ Age: 22 ± 2 y	I: drink w/sucralose (4 g) C: drink w/sucrose (31 g) Drinks served w/a meal *Non-isoenergetic condition*	Diff. *b*/*w* groups ^a^: 24 h AUC_FAT_: I ↑* than C	Diff. *b*/*w* groups: 24 h AUC_CHO_: I↓*** than C	Diff. *b*/*w* groups: 24 h AUC_EE_: I↓*** than C 8 h DIT: I ↑* than C
Chern, 2019 [62]	n: 11 (f = 3, m = 8) BMI: 24.9 ± 6.1 kg/m^2^ Age: 25.0 ± 4.7 y	I_1_: jelly w/sucralose (0.12 g) I_2_: jelly w/sucralose (0.12 g)/maltodextrin (50 g) C: jelly w/sucrose (50 g) *I*_1_* vs. I*_2_ *and C: non-isoenergetic condition* *I*_2_ *vs. C: isoenergetic*	Diff. *b*/*w* groups: 1.5 h AUC_FAT_: NS b/w I_1_ and I_2_ I_1_ and I_2_ ↑* than C	Diff. *b*/*w* groups: 1.5 h AUC_CHO_: I_1_ ↓*** I_2_ I_1_ and I_2_ ↓*** than C Time-meal I/A_CHO_: overall sign.*** ^b^	Diff. *b*/*w* groups: 1.5 h AUC_EE_: I_1_ ↓* I_2_ and C NS b/w I_2_ and C Time-meal I/A_EE_: NS b/w I_1_, I_2_ and C
Eckstein, 2021 [36]	n: 15 (f = 5, m = 10) BMI: 23.7 ± 1.7 kg/m^2^ Age: 25.4 ± 2.5 y	I: drink w/sucralose (0.2 g) C_1_: drink w/glucose (1 g/kg body mass) C_2_: drink w/fructose (1 g/kg body mass) C_3_: drink w/fructose/glucose (1 g/kg body mass) *Non-isoenergetic condition*	Diff. *b*/*w* groups: PEAK RQ: overall sign.** ^b^ END ^c^ RQ: overall sign.* ^b^	Diff. *b*/*w* groups: PEAK_CHO_: overall sign.** ^b^ END^c^_CHO_: NS	
Pearson, 2021 [65]	n: 8 m BMI no info, height = 176.9 ± 6.0 cm weight = 82.4 ± 6.2 kg Age: 22 ± 1.8 y	I: drink w/aspartame ^d^ C_1_: drink w/water ^d^ C_2_: drink w/sucrose ^d^ (approx. 60 g sucrose) Drinks served w/a meal *I and C*_1_*: isoenergetic condition* *I and C*_2_: non-isoenergetic condition	Diff. *b*/*w* groups: 3 h AUC_FAT_: I NS vs. C_1_ I ↑* than C_2_ Time-meal I/A_FAT_: I ↑ than C_2_ at 10 min**, 2 h** and 3 h** I ↓** than C_1_ at 10 min	Diff. *b*/*w* groups: 3 h AUC_CHO_: I NS vs. C_1_ I ↓* than C_2_ Time-meal I/A_CHO_: I ↓ than C_2_ at 10 min**, 2 h* and 3 h* I ↑** than C_1_ at 10 min	Diff. *b*/*w* groups: 3 h AUC_EE_: I NS vs. C_2_ I ↑* than C_1_ Time-meal I/A_EE_: I NS vs. C_2_ I ↑ than C_1_ at 10 min* and 1 h**
Prat-Larquemin, 2000 [64]	n: 24 m BMI: 20.4 ± 0.4 kg/m^2^ Age: 23.2 ± 0.5 y	I: cheese w/aspartame (0.27 g)/maltodextrin (90 g) C_1_: cheese w/sucrose (90 g) C_2_: cheese w/maltodextrin (90 g) *Isoenergetic condition*			Diff. *b*/*w* groups: 5 h DIT: NS b/w I, C_1_, C_2_ Time-meal I/A_EE_: 0.5–1, 1.5–2 h and peak: NS *b*/*w* I and C_2_ I and C_2_ ↓ * than C_1_
Mourão, 2004 [37]	n: 26 m, 13 w/NW, 13 w/OW NW: BMI: 23.4 ± 0.5 kg/m^2^ Age: 25.0 ± 1.6 y OW: BMI: 29.3 ± 0.6 kg/m^2^ Age: 30.0 ± 1.9 y	I: meal w/sucralose (4 g) C: meal w/sucrose (21 g) *Isoenergetic condition*	Diff. b/w groups: 4 h g/min_FAT_: NS b/w I and C	Diff. b/w groups: 4 h g/min_CHO_: I ↑* than C	Diff. b/w groups: 2 h AUC_EE_: NS b/w I and C 0.5 h AUC_EE_ 4 h after meal consumption: I ↑* than C 3 h DIT: I ↑* than C
Kimura, 2017 [63]	n: 13 (f = 8, m = 5) BMI: 20.9 ± 0.7 kg/m^2^ Age: 35.7 ± 2.1 y	I: drink w/d-allulose (5 g) C: drink w/aspartame (10 g) Drinks served before a meal *Isoenergetic condition*	Diff. *b*/*w* groups: 4 h AUC_FAT_: I ↑* than C Time-meal I/A_FAT_: I ↑** than C at 1.5 h	Diff. *b*/*w* groups: 4 h AUC_CHO_: I ↓* than C Time-meal I/A_CHO_: I ↓ than C at 1.5*, 3.5 h** and 4 h**	Diff. *b*/*w* groups: 4 h AUC_EE_: NS *b*/*w* I and C
Veldhuizen, 2017 [51]	n: 18 (f = 12, m = 6) BMI: 21.9 ± 2.1 kg/m^2^ Age: 24.1 ± 3.6 y	I: drink w/sucralose/maltodextrin sweetened to be equivalent to a 315 kJ sucrose drink C: drink with sucralose/maltodextrin sweetened to be equivalent to a 472.5 kJ sucrose drink *Isoenergetic condition*			Diff. *b*/*w* groups: DIT 30 min: I ↓* than C
**Longer-term study > 1 day**					
Sørensen ^e^, 2014 [52]	n: 22 NCS: f = 12, m = 2 Sucrose: f = 8, m = 2 NCS: BMI: 27.3 ± 2.5 kg/m^2^ Age: 35.2 ± 12.4 y Sucrose: BMI: 28.7 ± 2.3 kg/m^2^ Age: 35.3 ± 9.8 y	I: diet w/NCS ^f^ C: diet w/sucrose (125–175 g/d) *Non-isoenergetic condition*	Week-diet I/A_FAT_ ^a^: I ↑*** than C at week 10	Week-diet I/A_CHO_: I ↓*** than C at week 10	Diff. *b*/*w* groups (week 10): 24 h AUC_EE_: NS b/w I and C BMR: NS *b*/*w* I and C 4 h DIT: NS b/w I and C Week-time-diet I/A_EE_: I ↓** than C at 11.00 a.m. in week 10

^a^ Substrate oxidation and EE were measured through indirect calorimetry via a respiration chamber. ^b^ It was not specified which of the groups differed. ^c^ END is RQ/CHO measured after 2 h. ^d^ 20oz = approximately 600 mL Diet-Coke, Coca-Cola, or water, was served. Since no specific information about the dose for sucrose and aspartame was reported, we estimated the amount of sucrose based on this information. ^e^ This study had a parallel design. ^f^ No information about dose provided. Abbreviations: approx., approximately; AUC, area under the curve; BMI, body mass index; BMR, basal metabolic rate; *b*/*w*, between; CHO, carbohydrate oxidation; C, control; diff., difference; DIT, diet-induced thermogenesis; EE, energy expenditure; FAT, fat oxidation; f, female; I/A, interaction; I, intervention; m, male; n, number; NS, no(n) significan(t)ce; NW, normal weight; OW, overweight; RQ = respiratory quotient; sign., significance; vs., versus; w/, with; ↑, significantly higher; ↓, significantly lower; *, level of significance: * *p* < 0.05, ** *p* < 0.01, *** *p* < 0.001.

**Table 2:** Low-caloric sweeteners—fat oxidation, carbohydrate oxidation, and energy expenditure. Unless otherwise stated, all outcomes were measured in a respiration chamber, and the studies had a randomized crossover design.

First Author, Year	Population Characteristics	Intervention and Control	Results		
			FAT	CHO	EE and DIT
**Acute studies ≤ 1 day**					
Felber, 1987 [53]	n: 8 (sex = no info) BMI: no info Age: 24 ± 2 y	I: drink w/maltitol (30 g) C: drink w/sucrose (30 g) *Non-isoenergetic condition*	Time-meal I/A_FAT_ ^a^: I ↑ at 0–0.5 h* and 0.5–1 h** than C I ↓ at 3–3.5 h* than C	Diff. *b*/*w* groups: 3 h iAUCc_HO_: I ↓* than C Time-meal I/A_CHO_: I ↓ at 0–1 h*** and 1–1.5 h* than C I ↑* at 3–3.5 h than C	
Müller-Hess ^b^, 1975 [54]	n: 7 (f = 3, m = 4) BMI: no info Age range: 20–30 y	I: drink w/xylitol (50 g) C: drink w/glucose (50 g) *Non-isoenergetic condition*		Diff. *b*/*w* groups: 2.5 h AUC_CHO_: I < 23% ^c^ than C	Diff. *b*/*w* groups: 2.5 h AUC_EE_: I < 46% ^c^ than C
Natah, 1997 [38]	n: 8 (m = all) BMI: 22.1 ± 0.5 kg/m^2^ Age: 25 ± 1 y	I_1_: drink w/xylitol (25 g) I_2_: drink w/lactitol (25 g) C: drink w/glucose (25 g) *Non-iosenergetic condition*	Diff. *b*/*w* groups ^a^: kJ/min_FAT_: I_1_ ↑* than C NS b/w I_2_ and C	Diff. *b*/*w* groups: kJ/min_CHO_: NS b/w I_1_, I_2_ and C	
Thiebaud, 1984 [55]	n: 10 (m = all) BMI: no info, height = 178.3 ± 4.8 cm, weight = 69.3 ± 4.2 kg Age: 26.1 ± 2.9 y	I: drink w/isomalt (36.1 g) C: drink w/sucrose (30 g) *Non-isoenergetic condition*	Diff. *b*/*w* groups ^a^: 6 h iAOC ^d^ _FAT_: I ↓** C	Diff. *b*/*w* groups: 6 h iAUC_CHO_: I ↓** C	
**Longer-term studies > 1 day**					
Buemann, 1998 [40]	n: 8 (f = 5, m = 3) f: BMI: no info, height = 169.0 ± 2.3 cm weight = 66.3 ± 2.9 kg Age: 26.2 ± 2.6 y m: BMI: no info, height = 183.7 ± 0.8 cm weight = 74.4 ± 3.9 kg Age: 25.0 ± 3.4 y	I: cake w/d-tagatose (30 g) C: cake w/sucrose (30 g) *Non-isoenergetic condition*	Diff. *b*/*w* groups: 24 h RQ day 1 and 15: NS *b*/*w* I and C Time-meal I/A RQ: NS *b*/*w* all 4 test days	Diff. *b*/*w* groups: 24 RQ day 1 and 15: NS *b*/*w* I and C Time-meal I/A RQ: NS *b*/*w* all 4 test days	Diff. *b*/*w* groups: 24 h AUC_EE_ day 1: NS *b*/*w* I and C 24 h AUC_EE_ day 15: NS *b*/*w* I and C Time-meal I/A_EE_: NS *b*/*w* all 4 test days
Sinaud, 2002 [56]	n: 9 (m = all) BMI: no info Age: no info	I_1_: diet w/high-polymer maltitol syrup (100 g/DM/d) I_2_: diet w/high-polymer (100 g/DM/d) C: diet w/dextrose (100 g/DM/d) *Non-isoenergetic condition*			Diff. *b*/*w* groups: 24 h AUC_EE_ day 32: I_1_ and 1_2_ ↑* than C
Van Es, 1986 [39]	n: 8 (f = 4, m = 4) Diet with sucrose: BMI: no info, weight = 64.6 ± 5.5 kg Diet with lactiol: BMI: no info, weight = 65.0 ± 5.5 kg Age: 22.3 ± 2.6 y	I: diet w/lactitol (50 g) C: diet w/sucrose (49 g) *Non-isoenergetic condition*	Diff. *b*/*w* groups: 24 ^e^ RQ day 8: I ↓* than C	Diff. *b*/*w* groups: 24 ^e^ RQ day 8: I ↓* than C	

^a^ Substrate oxidation and EE were measured through indirect calorimetry via a ventilated hood mask. ^b^ This study was a non-randomized crossover trial. ^c^ It is not stated in the article whether this difference is significant. ^d^ iAOC, how much fat oxidation is inhibited after the test drink. ^e^ RQ was not corrected for protein oxidation in this study. Abbreviation: AUC, area under the curve; BMI, body mass index; *b*/*w*, between; CHO, carbohydrate oxidation; C, control; diff., difference; DIT, diet-induced thermogenesis; DM, dry matter; EE, energy expenditure; FAT, fat oxidation; f, female; I/A, interaction; iAOC, incremental area over the curve; iAUC, incremental area under the curve; I, intervention; m, male; n, number; NS, no(n) significan(t)ce; RQ = respiratory quotient; w/, with; ↑, significantly higher; ↓, significantly lower; *, level of significance: * *p* < 0.05, ** *p* < 0.01, *** *p* < 0.001.

**Table 3:** Non- and low-caloric sweeteners—norepinephrine, epinephrine, and dopamine. Unless otherwise stated, all outcomes were measured via blood samples, and the studies had a randomized crossover design.

First Author, Year	Population Characteristics	Intervention and Control	Results		
			Norepinephrine	Epinephrine	Dopamine
**Acute studies ≤ 1 day**					
Jones ^a^, 1995 [57]	n: 6 (f = 3, m = 3) BMI: no info Age: 10 ± 3 y	I: drink w/aspartame and acesulfame potassium ^b^ C: drink w/glucose (1.75 gm/kg body weight) *Non-isoenergetic condition*		Time-meal I/A: I ↓* at 4 and 4.5 h than C	
Tse, 1983 [59]	n: 10 (f = 2, m = 8) BMI range: 51.6–82.5 kg Age: 24 y	I_1_: drink w/xylose (62.5 g) I_2_: drink w/mannitol (20 g) C: drink w/water *Non-isoenergetic condition*	Diff. *b*/*w* groups: 5 h conc. NS *b*/*w* I_1_, I_2_ vs. C	Diff. *b*/*w* groups: 5 h conc. NS *b*/*w* I_1_ and C 5 h conc. NS *b*/*w* I_2_ and C	
Schiffman, 1987 [58]	n: 40 (f = 28, m = 12) BMI: no info, height = 166.8 ± 1.37 cm weight = 76.9 ± 3.5 kg Age: 33.5 ± 1.9 y	I: capsules w/aspartame (10 mg/kg body weight) C: capsules w/microcrystalline cellulose Meals were served during the test days *Isoenergetic condition*	Diff. *b*/*w* groups: 9 a.m., 11 a.m., 4 p.m., and at times of adverse events conc.: NS *b*/*w* I and C	Diff. *b*/*w* groups: 9 a.m., 11 a.m., 4 p.m., and at times of adverse events conc.: NS *b*/*w* I and C	
**Longer-term study > 1 day**					
Shaywitz, 1994 [60]	n: 15 (f = 4, m = 11) BMI: no info, weight = 35.4 ± 12.6 kg Age: 8.9 ± 2.5 y	I: capsules w/aspartame (34.7 mg/kg body weight) C: capsules w/microcrystalline cellulose *Isoenergetic condition*	Diff. b/w groups ^c^: day 9: NS *b*/*w* I and C	Diff. *b*/*w* groups: day 9: NS b/w I and C	Diff. *b*/*w* groups: day 9: NS

^a^ This study is a non-randomized controlled crossover trial. ^b^ No information about dose provided. ^c^ Concentration of catecholamines was measured in urine in this study. Abbreviation: BMI, body mass index; *b*/*w*, between; conc., concentration; C, control; diff., difference; f, female; I/A, interaction; I, intervention; m, male; n, number; NS, no(n) significan(t)ce; vs., versus; w/, with; ↓, significantly lower; *, level of significance: * *p* < 0.05.

## Data Availability

Not applicable.

## Supplementary Materials

The following supporting information can be downloaded at: https://www.mdpi.com/article/10.3390/nu15122711/s1, Table S1: Databases and database-specific filters; Table S2: Search terms; Table S3: Non-caloric sweeteners—substrate oxidation and energy expenditure; Table S4: Low-caloric sweeteners—substrate oxidation and energy expenditure; Table S5: Non- and low-caloric sweeteners—catecholamines.

## References

[1] World Health Organization (2018). Noncommunicable Diseases Country Profiles 2018.

[2] World Health Organization (WHO) (2015). Guideline: Sugars Intake for Adults and Children.

[3] Msomi N.Z., Erukainure O.L., Islam M.S. (2021). Suitability of Sugar Alcohols as Antidiabetic Supplements: A Review. J. Food Drug Anal..

[4] Rice T., Zannini E., Arendt E.K., Coffey A. (2020). A Review of Polyols–Biotechnological Production, Food Applications, Regulation, Labeling and Health Effects. Crit. Rev. Food Sci. Nutr..

[5] Chattopadhyay S., Raychaudhuri U., Chakraborty R. (2014). Artificial Sweeteners—A Review. J. Food Sci. Technol..

[6] Martí N., Funes L.L., Saura D., Micol V. (2008). An Update on Alternative Sweeteners. Int. Sugar J..

[7] Magnuson B.A., Carakostas M.C., Moore N.H., Poulos S.P., Renwick A.G. (2016). Biological Fate of Low-Calorie Sweeteners. Nutr. Rev..

[8] Livesey G. (2003). Health Potential of Polyols as Sugar Replacers, with Emphasis on Low Glycaemic Properties. Nutr. Res. Rev..

[9] Mattes R.D., Popkin B.M. (2009). Nonnutritive Sweetener Consumption in Humans: Effects on Appetite and Food Intake and Their Putative Mechanisms. Am. J. Clin. Nutr..

[10] Grembecka M. (2015). Sugar Alcohols-Their Role in the Modern World of Sweeteners: A Review. Eur. Food Res. Technol..

[11] Ashwell M., Gibson S., Bellisle F., Buttriss J., Drewnowski A., Fantino M., Gallagher A.M., de Graaf K., Goscinny S., Hardman C.A. (2020). Expert Consensus on Low-Calorie Sweeteners: Facts, Research Gaps and Suggested Actions. Nutr. Res. Rev..

[12] Romo-Romo A., Aguilar-Salinas C.A., Gómez-Díaz R.A., Brito-Córdova G.X., Gómez-Velasco D.V., López-Rocha M.J., Almeda-Valdés P. (2017). Non-Nutritive Sweeteners: Evidence on Their Association with Metabolic Diseases and Potential Effects on Glucose Metabolism and Appetite. Rev. Investig. Clin..

[13] Dybing E., Doe J., Groten J., Kleiner J., O’Brien J., Renwick A.G., Schlatter J., Steinberg P., Tritscher A., Walker R. (2002). Hazard Characterisation of Chemicals in Food and Diet: Dose Response, Mechanisms and Extrapolation Issues. Food Chem. Toxicol..

[14] World Health Organization (WHO) (2022). Health Effects of the Use of Non-Sugar Sweeteners: A Systematic Review and Meta-Analysis.

[15] Zhao X., Yan J., Chen K., Song L., Sun B., Wei X. (2018). Effects of Saccharin Supplementation on Body Weight, Sweet Receptor MRNA Expression and Appetite Signals Regulation in Post-Weanling Rats. Peptides.

[16] Suez J., Korem T., Zeevi D., Zilberman-Schapira G., Thaiss C.A., Maza O., Israeli D., Zmora N., Gilad S., Weinberger A. (2014). Artificial Sweeteners Induce Glucose Intolerance by Altering the Gut Microbiota. Nature.

[17] Azad M.B., Abou-Setta A.M., Chauhan B.F., Rabbani R., Lys J., Copstein L., Mann A., Jeyaraman M.M., Reid A.E., Fiander M. (2017). Nonnutritive Sweeteners and Cardiometabolic Health: A Systematic Review and Meta-Analysis of Randomized Controlled Trials and Prospective Cohort Studies. CMAJ.

[18] Romo-Romo A., Aguilar-Salinas C.A., Brito-Cordova G.X., Valentin D.V., Almeda-Valdes P., Diaz R.A.G. (2016). Effects of the Non-Nutritive Sweeteners on Glucose Metabolism and Appetite Regulating Hormones: Systematic Review of Observational Prospective Studies and Clinical Trials. PLoS ONE.

[19] Lu C.Y. (2009). Observational Studies: A Review of Study Designs, Challenges and Strategies to Reduce Confounding. Int. J. Clin. Pract..

[20] Normand M., Ritz C., Mela D., Raben A. (2021). Low-Energy Sweeteners and Body Weight: A Citation Network Analysis. BMJ Nutr. Prev. Health.

[21] World Health Organization (WHO) (2023). Guideline: Use of Non-Sugar Sweeteners.

[22] Payne A.N., Chassard C., Lacroix C. (2012). Gut Microbial Adaptation to Dietary Consumption of Fructose, Artificial Sweeteners and Sugar Alcohols: Implications for Host-Microbe Interactions Contributing to Obesity. Obes. Rev..

[23] Nettleton J.E., Reimer R.A., Shearer J. (2016). Reshaping the Gut Microbiota: Impact of Low Calorie Sweeteners and the Link to Insulin Resistance?. Physiol. Behav..

[24] Pearlman M., Obert J., Casey L. (2017). The Association Between Artificial Sweeteners and Obesity. Curr. Gastroenterol. Rep..

[25] Burke M.V., Small D.M. (2015). Physiological Mechanisms by Which Non-Nutritive Sweeteners May Impact Body Weight and Metabolism. Physiol. Behav..

[26] Pang M.D., Goossens G.H., Blaak E.E. (2021). The Impact of Artificial Sweeteners on Body Weight Control and Glucose Homeostasis. Front. Nutr..

[27] O’Connor D., Pang M., Castelnuovo G., Finlayson G., Blaak E., Gibbons C., Navas-Carretero S., Almiron-Roig E., Harrold J., Raben A. (2021). A Rational Review on the Effects of Sweeteners and Sweetness Enhancers on Appetite, Food Reward and Metabolic/Adiposity Outcomes in Adults. Food Funct..

[28] McGlynn N.D., Khan T.A., Wang L., Zhang R., Chiavaroli L., Au-Yeung F., Lee J.J., Noronha J.C., Comelli E.M., Blanco Mejia S. (2022). Association of Low- and No-Calorie Sweetened Beverages as a Replacement for Sugar-Sweetened Beverages with Body Weight and Cardiometabolic Risk: A Systematic Review and Meta-Analysis. JAMA Netw. Open.

[29] Zhang R., Noronha J.C., Khan T.A., Mcglynn N., Back S., Grant S.M., Kendall C.W.C., Sievenpiper J.L. (2023). The Effect of Non-Nutritive Sweetened Beverages on Postprandial Glycemic and Endocrine Responses: A Systematic Review and Network Meta-Analysis. Nutrients.

[30] Rogers P.J., Appleton K.M. (2021). The Effects of Low-Calorie Sweeteners on Energy Intake and Body Weight: A Systematic Review and Meta-Analyses of Sustained Intervention Studies. Int. J. Obes..

[31] Rogers P.J., Hogenkamp P.S., De Graaf C., Higgs S., Lluch A., Ness A.R., Penfold C., Perry R., Putz P., Yeomans M.R. (2016). Does Low-Energy Sweetener Consumption Affect Energy Intake and Body Weight? A Systematic Review, Including Meta-Analyses, of the Evidence from Human and Animal Studies. Int. J. Obes..

[32] Higgins J.P.T., Lasserson T., Chandler J., Tovey D., Thomas J., Flemyng E., Churchill R. (2021). Standards for the conduct of new Cochrane Intervention Reviews. Methodological Expectations of Cochrane Intervention Reviews.

[33] Shamseer L., Moher D., Clarke M., Ghersi D., Liberati A., Petticrew M., Shekelle P., Stewart L.A. (2015). Preferred Reporting Items for Systematic Review and Meta-Analysis Protocols (PRISMA-P) 2015: Elaboration and Explanation. BMJ.

[34] Bland J.M., Altman D.G. (2011). Comparisons against Baseline within Randomised Groups Are Often Used and Can Be Highly Misleading. Trials.

[35] (2022). Covidence Systematic Review Software. Verit. Heal. Innov. Melbourne, Aust. www.covidence.org.

[36] Eckstein M.L., Brockfeld A., Haupt S., Schierbauer J.R., Zimmer R.T., Wachsmuth N., Zunner B., Zimmermann P., Obermayer-Pietsch B., Moser O. (2021). Acute Metabolic Responses to Glucose and Fructose Supplementation in Healthy Individuals: A Double-Blind Randomized Crossover Placebo-Controlled Trial. Nutrients.

[37] Mourão D.M., Monteiro J.B.R., Hermsdorff H.H.M., Lelte M.C.T. (2004). Effect of Sucrose and Sweetener on Appetite Sensation and Energy Expenditure in Normal Weight and Overweight Subjects. Rev. Bras. Nutr. Clin..

[38] Natah S.S., Hussien K.R., Tuominen J.A., Koivisto V.A. (1997). Metabolic Response to Lactitol and Xylitol in Healthy Men. Am. J. Clin. Nutr..

[39] Van Es A.J.H., De Groot L., Vogt J.E. (1986). Energy Balances of Eight Volunteers Fed on Diets Supplemented with Either Lac Ti To1 or Saccharose. Br. J. Nutr..

[40] Buemann B., Toubro S., Astrup A. (1998). D-Tagatose, a Stereoisomer of d-Fructose, Increases Hydrogen Production in Humans without Affecting 24-Hour Energy Expenditure or Respiratory Exchange Ratio. J. Nutr..

[41] Higgins J.P.T., Savović E., Page M.J., Sterne J.A.C. Revised Cochrane Risk of Bias Tool for Randomized Trials (RoB 2) Additional Considerations for Crossover Trials. https://www.riskofbias.info/welcome/rob-2-0-tool/rob-2-for-crossover-trials.

[42] Higgins J., Sterne J., Savović J., Page M., Hrobjartsson A., Bourton I., Reeves B., Eldridge S. A Revised Cochrane Risk of Bias Tool for Randomized Trials. https://sites.google.com/site/riskofbiastool/welcome/rob-2-0-tool/current-version-of-rob-2?authuser=0.

[43] Sterne J.A.C., Hernán M.A., Reeves B.C., Savović J., Berkman N.D., Viswanathan M., Henry D., Altman D.G., Ansari M.T., Boutron I. (2016). ROBINS-I: A tool for assessing risk of bias in non-randomized studies of interventions. BMJ.

[44] Sterne J.A.C., Savović J., Page M.J., Elbers R.G., Blencowe N.S., Boutron I., Cates C.J., Cheng H.-Y., Corbett M.S., Eldridge S.M. (2019). RoB 2: A Revised Tool for Assessing Risk of Bias in Randomised Trials. BMJ.

[45] Campbell M., McKenzie J.E., Sowden A., Katikireddi S.V., Brennan S.E., Ellis S., Hartmann-Boyce J., Ryan R., Shepperd S., Thomas J. (2020). Synthesis without Meta-Analysis (SWiM) in Systematic Reviews: Reporting Guideline. BMJ.

[46] Guyatt G.H., Oxman A.D., Vist G.E., Kunz R., Falck-Ytter Y., Alonso-Coello P., Schünemann H.J. (2009). GRADE: An Emerging Consensus on Rating Quality of Evidence and Strength of Recommendations. Chin. J. Evid.-Based Med..

[47] Goldet G., Howick J. (2013). Understanding GRADE: An Introduction. J. Evid. Based Med..

[48] Grupp U., Siebert G. (1978). Metabolism of Hydrogenated Palatinose, an Equimolar Mixture of Alpha-D-Glucopyranosido-1,6-Sorbitol and Alpha-D-Glucopyranosido-1,6-Mannitol. Res. Exp. Med..

[49] Melanson K.J., Westerterp-Plantenga M.S., Campfield L.A., Saris W.H.M. (1999). Blood Glucose and Meal Patterns in Time-Blinded Males, after Aspartame, Carbohydrate, and Fat Consumption, in Relation to Sweetness Perception. Br. J. Nutr..

[50] Astrup A., Bulow J., Christensen N.J., Madsen J., Quaade F. (1986). Facultative Thermogenesis Induced by Carbohydrate: A Skeletal Muscle Component Mediated by Epinephrine. Am. J. Physiol. Metab..

[51] Veldhuizen M.G., Babbs R.K., Patel B., Fobbs W., Kroemer N.B., Garcia E., Yeomans M.R., Small D.M. (2017). Integration of Sweet Taste and Metabolism Determines Carbohydrate Reward. Curr. Biol..

[52] Sørensen L.B., Vasilaras T.H., Astrup A., Raben A. (2014). Sucrose Compared with Artificial Sweeteners: A Clinical Intervention Study of Effects on Energy Intake, Appetite, and Energy Expenditure after 10 Wk of Supplementation in Overweight Subjects. Am. J. Clin. Nutr..

[53] Felber J.P., Tappy L., Vouillamoz D., Randin J.P., Jéquier E. (1987). Comparative Study of Maltitol and Sucrose by Means of Continuous Indirect Calorimetry. JPEN J. Parenter. Enteral Nutr..

[54] Müller-Hess R., Geser C.A., Bonjour J.P., Jéquier E., Felber J.P. (1975). Effects of Oral Xylitol Administration on Carbohydrate and Lipid Metabolism in Normal Subjects. Infusionsther. Klin. Ernahr..

[55] Thiébaud D., Jacot E., Schmitz H., Spengler M., Felber J.P. (1984). Comparative Study of Isomalt and Sucrose by Means of Continuous Indirect Calorimetry. Metabolism.

[56] Sinaud S., Montaurier C., Wils D., Vernet J., Brandolini M., Bouteloup-Demange C., Vermorel M. (2002). Net Energy Value of Two Low-Digestible Carbohydrates, Lycasin (R) HBC and the Hydrogenated Polysaccharide Fraction of Lycasin (R) HBC in Healthy Human Subjects and Their Impact on Nutrient Digestive Utilization. Br. J. Nutr..

[57] Jones T.W., Borg W.P., Boulware S.D., McCarthy G., Sherwin R.S., Tamborlane W. (1995). V Enhanced Adrenomedullary Response and Increased Susceptibility to Neuroglycopenia: Mechanisms Underlying the Adverse Effects of Sugar Ingestion in Healthy Children. J. Pediatr..

[58] Schiffman S.S., Buckley C.E., Sampson H.A., Massey E.W., Baraniuk J.N., Follett J.V., Warwick Z.S. (1987). Aspartame and Susceptibility to Headache. N. Engl. J. Med..

[59] Tse T.F., Clutter W.E., Shah S.D., Miller J.P., Cryer P.E. (1983). Neuroendocrine Responses to Glucose Ingestion in Man. Specificity, Temporal Relationships, and Quantitative Aspects. J. Clin. Investig..

[60] Shaywitz B.A., Sullivan C.M., Anderson G.M., Gillespie S.M., Sullivan B., Shaywitz S.E. (1994). Aspartame, Behavior, and Cognitive Function in Children with Attention Deficit Disorder. Pediatrics.

[61] Casperson S.L., Hall C., Roemmich J.N. (2017). Postprandial Energy Metabolism and Substrate Oxidation in Response to the Inclusion of a Sugar- or Non-Nutritive Sweetened Beverage with Meals Differing in Protein Content. BMC Nutr..

[62] Chern C., Tan S.-Y. (2019). Energy Expenditure, Carbohydrate Oxidation and Appetitive Responses to Sucrose or Sucralose in Humans: A Pilot Study. Nutrients.

[63] Kimura T., Kanasaki A., Hayashi N., Yamada T., Iida T., Nagata Y., Okuma K. (2017). D-Allulose Enhances Postprandial Fat Oxidation in Healthy Humans. Nutrition.

[64] Prat-Larquemin L., Oppert J.M., Bellisle F., Guy-Grand B. (2000). Sweet Taste of Aspartame and Sucrose: Effects on Diet-Induced Thermogenesis. Appetite.

[65] Pearson R.C., Green E.S., Olenick A.A., Jenkins N.T. (2021). Comparison of Aspartame- and Sugar-Sweetened Soft Drinks on Postprandial Metabolism. Nutr. Health.

[66] Hue L., Taegtmeyer H. (2009). The Randle Cycle Revisited: A New Head for an Old Hat. Am. J. Physiol.-Endocrinol. Metab..

[67] Ludwig D.S. (2002). The Glycemic Index Physiological Mechanisms Relating to Obesity, Diabetes, and Cardiovascular Disease. JAMA.

[68] Melzer K. (2011). Carbohydrate and Fat Utilization during Rest and Physical Activity. e-SPEN.

[69] Weyer C., Snitker S., Rising R., Bogardus C., Ravussin E. (1999). Determinants of Energy Expenditure and Fuel Utilization in Man: Effects of Body Composition, Age, Sex, Ethnicity and Glucose Tolerance in 916 Subjects. Int. J. Obes..

[70] Miles-Chan J.L., Dulloo A.G., Schutz Y. (2015). Fasting Substrate Oxidation at Rest Assessed by Indirect Calorimetry: Is Prior Dietary Macronutrient Level and Composition a Confounder. Int. J. Obes..

[71] Schutz Y. (1995). Abnormalities of Fuel Utilization as Predisposing to the Development of Obesity in Humans. Obes. Res..

[72] Cummings J.H., Roberfroid M.B., Andersson H., Barth C., Ferro-Luzzi A., Ghoos Y., Gibney M., Hermonsen K., James W.P.T., Korver O. (1997). A New Look at Dietary Carbohydrate: Chemistry, Physiology and Health. Eur. J. Clin. Nutr..

[73] Laffitte A., Neiers F., Briand L. (2014). Functional Roles of the Sweet Taste Receptor in Oral and Extraoral Tissues. Curr. Opin. Clin. Nutr. Metab. Care.

[74] Prather J.H., William L. (1993). Effects of Colonic Fermentation on Respiratory Gas Exchanges Glucose Load in Man. Metabolism.

[75] Westerterp K.R. (2004). Diet Induced Thermogenesis. Nutr. Metab..

[76] Diaz E.O., Prentice A.M., Goldberg G.R., Murgatroyd P.R., Coward W.A. (1992). Metabolic Response to Experimental Overfeeding in Lean and Overweight Healthy Volunteers. Am. J. Clin. Nutr..

[77] Reed G.W., Hill J.O. (1996). Measuring the Thermic Effect of Food. Am. J. Clin. Nutr..

[78] Canfora E.E., Van Der Beek C.M., Jocken J.W.E., Goossens G.H., Holst J.J., Olde Damink S.W.M., Lenaerts K., Dejong C.H.C., Blaak E.E. (2017). Colonic Infusions of Short-Chain Fatty Acid Mixtures Promote Energy Metabolism in Overweight/Obese Men: A Randomized Crossover Trial. Sci. Rep..

[79] Procházková N., Falony G., Dragsted L.O., Licht T.R., Raes J., Roager H.M. (2023). Advancing Human Gut Microbiota Research by Considering Gut Transit Time. Gut.

[80] Tarini J., Wolever T.M.S. (2010). The Fermentable Fibre Inulin Increases Postprandial Serum Short-Chain Fatty Acids and Reduces Free-Fatty Acids and Ghrelin in Healthy Subjects. Appl. Physiol. Nutr. Metab..

